# Cognitive Flexibility and Clinical Severity in Eating Disorders

**DOI:** 10.1371/journal.pone.0020462

**Published:** 2011-06-15

**Authors:** Kate Tchanturia, Amy Harrison, Helen Davies, Marion Roberts, Anna Oldershaw, Michiko Nakazato, Daniel Stahl, Robin Morris, Ulrike Schmidt, Janet Treasure

**Affiliations:** 1 Institute of Psychiatry, Psychological Medicine, King's College London, London, United Kingdom; 2 Department of Biostatistics, Institute of Psychiatry, King's College London, London, United Kingdom; 3 Department of Psychology, Institute of Psychiatry, King's College London, London, United Kingdom; University of Granada, Spain

## Abstract

**Objectives:**

The aim of this study was to explore cognitive flexibility in a large dataset of people with Eating Disorders and Healthy Controls (HC) and to see how patient characteristics (body mass index [BMI] and length of illness) are related to this thinking style.

**Methods:**

A dataset was constructed from our previous studies using a conceptual shift test - the Brixton Spatial Anticipation Test. 601 participants were included, 215 patients with Anorexia Nervosa (AN) (96 inpatients; 119 outpatients), 69 patients with Bulimia Nervosa (BN), 29 Eating Disorder Not Otherwise Specified (EDNOS), 72 in long-term recovery from AN (Rec AN) and a comparison group of 216 HC.

**Results:**

The AN and EDNOS groups had significantly more errors than the other groups on the Brixton Test. In comparison to the HC group, the effect size decrement was large for AN patients receiving inpatient treatment and moderate for AN outpatients.

**Conclusions:**

These findings confirm that patients with AN have poor cognitive flexibility. Severity of illness measured by length of illness does not fully explain the lack of flexibility and supports the trait nature of inflexibility in people with AN.

## Introduction

In neuropsychological studies of anorexia nervosa (AN), two main characteristics have emerged across different studies. One is poor flexibility (set-shifting) (for a systematic review, see Roberts et al. 2007 [1]) and the other is weak central coherence (for a systematic review, see Lopez et al. 2008 [2]). For bulimia nervosa (BN) the cognitive signature is less clear [3]. A recent review on neurocognition and eating disorders (EDs) [4] has highlighted difficulties in the appraisal of the literature due to small sample sizes and inconsistencies in methodology. This problem is exacerbated when the number of variables used to investigate neuropsychological functioning are substantial.

Nevertheless, in the last decade, interest in neuropsychology in the ED field has grown and there is a need for more clarity regarding findings [4]. This study aimed to take a focused approach to the largest database in EDs, to our knowledge, and join together all the available published and unpublished data from different studies in our department to address the following questions: (1) Is poor cognitive flexibility present in AN and BN patients? and (2) Is severity of illness associated with poorer performance in a cognitive flexibility test? These questions are important in understanding aspects of the illness, such as differences in cognitive characteristics between diagnostic categories and whether cognitive flexibility is a state or trait phenomenon, or a marker of severity. To address these questions, we focused on the Brixton Spatial Anticipation Test, which was used in a number of studies we have conducted using cohorts recruited from different settings: inpatients (published: [5], [6], [7]) outpatients (Harrison et al. [submitted]; Schmidt et al. [submitted] and Davies et al. [unpublished]), mixed inpatient and outpatient (published: [8]), and a mixed outpatient and community sample (published: [9]). The advantage of joining these various datasets together was to provide greater statistical power and a range of severity of illness.

### Hypotheses

The main hypothesis was that people with AN would show poorer performance on set-shifting, as measured using the Brixton Spatial Anticipation Test. We also predicted that chronic cases with a very low Body Mass Index (BMI: weight/height^2^) and longer history of illness (measured in years) would perform less well (a greater number of errors) relative to mild cases and the comparison control group.

## Methods

All participants were recruited between 1998 and 2009 in our department. In line with the ethical standards laid down in the 1964 Declaration of Helsinki, all studies had received approval from the ethical committee of the South London and Maudsley (SLaM) NHS Foundation Trust. All participants provided written informed consent prior to their inclusion in the study.

ED patients were recruited from the SLaM Eating Disorders inpatient or outpatient units and diagnosed by experienced ED clinicians as fulfilling DSM-IV criteria for AN, BN or Eating Disorders Not Otherwise Specified (EDNOS). Our definition of EDNOS was based on that of Thomas et al. (2009) [10] and included people who fulfilled all criteria of AN, except the weight criterion; those who fulfilled all criteria for AN but still had menses; those without a fat phobia; and those with partial AN (defined as having features of AN but missing at least two of the four diagnostic criteria). The decision to include EDNOS patients is supported by a recent large meta-analysis of EDNOS which suggests that AN with a more lenient weight criterion and without amenorrhoea is very similar to AN as defined currently [10]. Patients from the EDNOS group were part of an ongoing randomised control trial (RCT) in our department. Healthy Control (HC) and Recovered AN (Rec AN) participants were recruited via advertisements in the local community, and through a circular email sent around to King's College London students and staff. Based on Bardone-Cone et al. 2010 [11], who state that a definition of recovery from an ED should include physical, behavioural, and psychological components, recovered participants were required to have a body mass index >18.5, restored menstruation for at least the past year and an absence of ED behaviours such as restriction or binge-purge symptoms during this period. These data were self-reported by participants. HC participants were excluded if they had any history of EDs, head injury or psychiatric illness. All participants were female and aged between 18 and 55 years old.

Cases from the final dataset were excluded if age, current BMI, length of illness or Brixton Test raw number of errors were missing. Additionally, ED cases were excluded if the BMI was higher than 26, and HCs were excluded if the BMI was lower than 19. Participants younger than 18 years of age were also excluded. Of those with AN, 96 were inpatients and 119 were outpatients at the time of assessment.

*The Brixton Spatial Anticipation Test* [12] is a concept (or ‘rule’) attainment task, which also incorporates switching between mental representations. The test consists of 56 trials and each has the same array of ten circles in a two by five matrix. On each trial, one circle is filled in with the colour blue. The position of this changes from trial to trial, with the participant having to determine a rule that governs the sequence of changes, predicting the location of the filled circle for the next trial. As the test progresses, the rule changes, requiring detection of the new rule. There is no time limit set for test completion, but the test requires around 5–10 minutes for administration. The total number of errors made on the test can be used to construct a scaled score. The Brixton Test is relatively quick and easy to administer in comparison to the Wisconsin Card Sorting Test, which is longer and more complex in nature. Common classes of errors made in this test are perseveration (repeating one's response), misapplication of a strategy, and random guesses.

In all studies presented in this paper, a semi-computerised version of the Brixton Test was used, where each trial is presented on a computer screen. The test administrator records responses in the normal fashion, but instead of turning pages as in the original test experimentation, a left mouse click is used to advance to the next trial. The original instructions and scoring sheets were used as described by Burgess and Shallice (1997) [12]. The outcome measure used is the number of errors made (maximum number is 54).

BMI was calculated on the day of testing by measuring the participant's weight and height. Duration of illness was calculated since onset of ED, as determined in the clinical interview.

Some, but not all studies included in this paper used the National Adult Reading Test (NART-R) [13] which requires the participant to read aloud two columns of phonetically irregular words (50) and the number of errors are recorded. This test has been used to provide an estimate of premorbid intellectual ability, as it is more resistant to brain damage and cognitive decline than other intellectual abilities. It correlates significantly with level of education and with full score IQ, as measured using the British version of the Wechsler Adult Intelligence Scale (WAIS-R) [14] (r = 0.77) [15]. A recent systematic review shows that the NART-R has been frequently used in eating disorder studies and people with AN included in studies have had above average IQ [16].

The data were inspected using histograms and Kolmogorov-Smirnov tests to assess assumptions of normal distribution. As the main outcome measure (the Brixton Test error) was normally distributed a one-way ANOVA was applied to analyse between group differences. Alpha was set at *p*<0.05 unless Bonferroni's correction for multiple comparisons was applied as indicated below. Cohen's *d* [17] (mean_1_-mean_2_/pooled standard deviation) was calculated to provide effect sizes for normally distributed data, with an effect size of <0.2 defined as small, <0.5 defined as medium and <0.8 defined as large [17]. To compare the data of different studies, we standardised them by calculating z scores using the mean and SD of the control group.

A general linear model regression analysis was used to assess the relationship between BMI and Brixton test scores within the five different groups with Brixton total scores as the dependent variable and BMI group and an interaction between group and BMI (which allows to model the effect of BMI on Brixton total within each group separately) as independent variables. Age was included as a potential confounder. A similar general linear model regression analysis was performed to assess the relationship between duration of illness and Brixton total score within the four clinical groups.

## Results

Table 1 provides clinical and demographic information for the five participant groups.

**Table 1:** Clinical and Demographic Characteristics of the Healthy Control (HC), Eating Disorder (AN, BN and EDNOS) and Recovered AN (REC AN) Groups.

	HCN = 216	ANN = 215	BNN = 69	EDNOSN = 29	RecANN = 72	Test Statistics
**Age**	27.0 (7.9)	26.9 (8.2)	27.7 (7.8)	26.5 (7.1)	30.2 (10.1)	F(4,597) = 2.5, p = 0.04
**BMI**	21.9 (1.8)	15.0 (1.7)	21.0 (2.1)	17.5 (1.5)	20.5 (1.6)	F(4,597) = 446.3, p = ≤0.001
**Duration of Illness**	N/A	10.7 (8.0)	8.9 (6.0)	6.1 (4.6)	Not included: 70% missing	F(4,381) = 48.5, p = ≤0.001
**IQ NART-R**	111.8 (7.2)	109.1 (8.7)	109.6 (6.9)	101.5 (11.5)	110.3 (8.3)	F(4,420) = 8.8, p = ≤0.001

HC = healthy control group; AN = anorexia nervosa group; BN = bulimia nervosa group; EDNOS = eating disorders not otherwise specified group; Rec AN = recovered AN group. IQ = intelligent quotient; NART = National Adult Reading Test. Test statistics are ANOVAs and descriptive statistics are means followed by standard deviations in parentheses. N/A = not applicable. BMI = Body mass index (weight/height^2^). 30% of data is missing on IQ (HC = 113; AN = 47; BN = 2; Rec = 15 because one of the studies did not include IQ in the research protocol.

There was a significant main effect of group for participants' age. Bonferroni corrected post-hoc tests revealed that the HC (p = 0.04) and AN groups (p = 0.03) were significantly younger than the recovered AN group. As expected, there was a significant main effect of group for participants' BMI, with the AN group having a lower BMI than all other groups (p≤0.001). Bonferroni corrected post-hoc tests revealed that those with EDNOS also had a significantly lower BMI than those with BN, Rec AN and HCs (p≤0.001). There was a significant main effect of group for length of illness. Bonferroni corrected post-hoc tests revealed that the AN group had a significantly longer length of illness than those with EDNOS and BN (p = 0.02). There was a significant main effect of group for NART-R estimated IQ scores. Bonferroni corrected post-hoc tests revealed that the EDNOS group had a significantly lower IQ than the other groups (p = ≤0.001).

The between group comparison for the Brixton Test errors is presented in Table 2. There was a main effect of group for the number of errors F(4,597) = 18.5, p = ≤0.001. AN and EDNOS groups had the highest number of errors on the Brixton Test with Bonferroni corrected post-hoc tests revealing significantly more errors made than the other groups (all differences: p≤0.001). Group differences in Brixton errors remained significant if IQ was included as a covariate in the whole analysis. It should be noted that only 70% of data had both Brixton and IQ scores. The IQ effect was not significant (p = .48).

**Table 2:** Number of Errors made on the Brixton Test for the Healthy Control (HC), Eating Disorder (AN, BN and EDNOS) and Recovered AN (Rec AN) Groups.

	HCN = 216	ANN = 215	BNN = 69	EDNOSN = 29	RecANN = 72
**Brixton Test total number of errors**	9.8 (4.2)^a^	12.8 (6.2)^b *^ *D* = 0.6*Z* = −.75N(51) = 23.7%	11.2 (4.3)^a^ *D* = 0.3*Z* = −.30N(10) = 14.5%	17.9 (7.5)^b*^ *D* = 1.7*Z* = −1.9N(15) = 51.7%	11.9 (6.5)^a^ *D* = 0.4*Z* = −.49N(13) = 18.0%

HC = healthy control group; AN = anorexia nervosa group; BN = bulimia nervosa group; EDNOS = eating disorders not otherwise specified; Rec AN = recovered AN group. The analysis is based on a One-Way ANOVA and descriptive statistics are means followed by standard deviations in parentheses. Where letters in superscript differ, group means are significantly different, same letters indicate non-significant differences between the groups. *D* = Cohen's *D* effect size for each group compared with HC. Z scores provided based on the control data; also provided is the number N and percentages of the participants from each group falling below the 10th percentile.

To establish the proportion of patients who might be considered to have poor cognitive flexibility the number and percentage within each patient group who fell below the 10th percentile based on the healthy control data was calculated (See Table 2). This highlights the substantially higher proportion in the EDNOS group.

Inpatients with AN (n = 96) had a significantly longer duration of illness than outpatients with AN (n = 119) t (204) = 4.14; p<0.001). Table 3, below, compares inpatients' and outpatients' performance on the Brixton Test. When compared to HC participants, the more severely ill AN inpatients had a large effect size, whereas the outpatients had a medium effect size.

**Table 3:** Number of Errors on the Brixton Test made by Inpatients and Outpatients with Anorexia Nervosa.

		Brixton Test Number of errors	Effect size in comparison to HCs
Total AN Group	(n = 215)	13.1 (6.4)	0.6
AN inpatients	(n = 96)	14.7 (7.1)	0.9
AN outpatients	(n = 119)	11.8 (5.3)	0.4

AN = anorexia nervosa. Data reported are means with standard deviations in parentheses. Inpatients (IP) age 27.7 (8.1); Outpatients (OP) 26.3 (8.2); IQ: IP- 109.3 (7.9); OP 108.7(9.5); BMI: IP 13.9 (1.5); OP15.9 (1.3). Duration of illness: IP-13.0 (8.1); OP 8.9 (7.4).

To assess the relationship between BMI and Brixton test scores within the five different groups a general linear model regression analysis was performed with Brixton test scores as the dependent variable and BMI and group as independent variables. To allow the effect of BMI to differ between groups, an interaction between BMI and group was included. Furthermore, age was included as a confounder because age correlated positively with Brixton test scores (Spearman rank correlation r = 0.15, p<0.01).

There was no significant effect of BMI (F(1,590) = 2.715, p = 0.1) or a significant interaction between BMI and group (F(4,590) = 2.124, p = 0.076). There was a positive effect of age on Brixton test scores (F(1,590) = 44.413, p<0.001, b = 0.117) and significant differences between group (F(4,590) = 3.113, p = 0.015.

A second general linear model regression analysis was done to assess the relationship between duration of illness and Brixton test scores within the four different clinical groups with Brixton test scores as the dependent variable and duration of illness and group as independent variables. To allow the effect to differ between groups, an interaction between duration of illness and group was included. Age was included as a confounder. The analyses revealed a significant main effect of duration of illness (F(1, 272) = 5.249, p = 0.023) and group (F(3,272) = 9.787, p<0.001) and a significant interaction between duration of illness and group (F(3,272) = 4.054, p = 0.008). An analysis of the interaction revealed that the duration of illness was not significant in AN (b = 0.005, SE(b) = 0.076, p = 0.949), recovered AN (b = 0.130, SE(b) = 0.165, p = 0.433) and BN (b = −0.183, SE(b) = 0.13, p = 0.16). There was a significant negative relationship between duration of illness and Brixton scores in the EDNOS group (b = −0851, SE(b) = 0.275, p = 0.002). The effect was significantly different than from all other groups (all p values<0.03). There was a positive effect of age on Brixton test scores (F(1,272) = 21.538, p<0.001, b = 0.299, SE(b) = 0.064).

Including BMI and the interaction between BMI and group in the model did not alter the main results. The interaction between group and duration of illness remained significant (F(3,268) = 4.52, p = 0.004) and there was no significant effect of BMI (F(1,268) = 2.183, p = 0.141) or of the interaction between BMI and group (F(3,268) = 1.713, p = 0.141).

## Discussion

This brief report aimed to combine a large number of published and unpublished data from our department in order to address the following questions: (1) Is poor cognitive flexibility, measured using the Brixton Test, present in AN and BN patients? and (2) Is severity of illness associated with poorer performance in this test? The main hypothesis, which was that people with AN would show poorer performance on set-shifting in comparison to controls was supported by the data, with a medium effect size. Those with EDNOS also had poorer set-shifting than HCs, with a large effect size. There was no significant difference between those with BN and HCs. Those who had recovered from AN had an intermediate profile and did not differ significantly from the acute AN group or HCs.

From the regression analysis, it seems that BMI is not related to Brixton Test errors for those with AN (therefore errors are not just a result of low weight). The regression analysis revealed that once age was controlled for, there was no association between duration of illness and Brixton Test errors for the AN group. These two results may suggest the trait nature of reduced flexibility, with poorer set-shifting in comparison to controls indicating a stable trait in those with AN that does not depend either on BMI or illness duration. This result is similar to previous reports suggesting set-shifting may be a biomarker for EDs [9], [18], [19]. There is new evidence suggesting that adolescents with restrictive AN have difficulties with set-shifting compared to age matched controls [20] which may support the trait nature of cognitive inflexibility. It could also mean that patients with AN who have strong trait inflexibility engage and respond to treatment poorly and therefore are the most severe group typically treated in an inpatient unit.

These findings support models of EDs, such as the Maudsley model [21], which proposes that an inflexible cognitive style may be involved in the maintenance of AN. The clinical differences between young people with BN and EDNOS (e.g. those with EDNOS have more depression and obsessive-compulsive symptoms) [21] may also be considered in terms of neurocognitive performance. From this study, those with EDNOS also had poorer set–shifting than HCs, with a large effect size and a significant proportion (52%) were shown to have low levels of cognitive flexibility. Unfortunately, EDNOS is the smallest group in this study, but given that there are no reports on this clinical group in terms of neuropsychological performance, we think it is important to report this data. There was no significant difference between those with BN and HCs, which is in line with a recent systematic review [3]. Those who had recovered from AN had an intermediate profile and did not differ significantly from individuals with AN or HCs.

### Limitations

The current study assesses cognitive flexibility using only one test and we were not able to control for medication effects due to missing data. It was not possible to control for IQ across the entire sample because there was 30% missing data from one of the studies included in this dataset.

### Clinical Implications

The findings may have important implications for the rehabilitation of people with AN and EDNOS. In a clinical setting, it may be possible to focus on increasing flexibility, by providing cognitive training, developing awareness of cognitive inflexibility and designing behavioural exercises to address inefficiencies in set shifting [7], [22].<

### Conclusions

These differences highlight the need for future studies to assess the differential cause of cognitive flexibility inefficiencies in eating disorders. These data do not support the transdiagnostic nature of EDs and suggests that we will need different therapeutic strategies/tools to work on cognition with different groups of ED patients.

## References

[1] Roberts M, Tchanturia K, Stahl D, Southgate L, Treasure J (2007). A systematic review and meta-analysis of set shifting ability in eating disorders.. Psych Med.

[2] Lopez C, Tchanturia K, Stahl D, Treasure J (2008). Central coherence in eating disorders: a systematic review.. Psych Med.

[3] Van den Eynde F, Guillaume S, Broadbent H, Campbell I, Schmidt U (2011). Neurocognition in bulimic eating disorders: a systematic review.. Acta Psych Scand.

[4] Zakzanis Z, Campbell Z, Polsinelli A (2010). Quantitative evidence for distinct cognitive impairment in anorexia nervosa and bulimia nervosa.. J Neuropsych.

[5] Tchanturia K, Brecelj M, Sanchez P, Morris R, Rabe-Hesketh S (2004). An examination of cognitive flexibility in eating disorders.. J Int Neuropsych Soc.

[6] Tchanturia K, Morris R, Brecelj M, Nikolau V, Treasure J (2004). Set Shifting in Anorexia Nervosa: An examination before and after weight gain, in full recovery and the relationship to childhood and adult OCDP traits.. J Psychiat Res.

[7] Tchanturia K, Davies H, Lopez C, Schmidt U, Treasure J (2008). Neuropsychological task performance before and after cognitive remediation in anorexia nervosa: A pilot case series.. Psych Med.

[8] Nakazato M, Tchanturia K, Schmidt U, Campbell I, Treasure J (2009). Brain derived neurotrophic factor (BDNF) and set-shifting in currently ill and recovered anorexia nervosa patients.. Psych Med.

[9] Roberts M, Tchanturia K, Treasure J (2010). Exploring the neurocognitive signature of poor set-shifting in anorexia and bulimia nervosa.. J Psychiat Res.

[10] Thomas J, Denisky S, St Germain S, Weigel T, Tangren C (2010). How do eating disorder specialist clinicians apply DSM-IV diagnostic criteria in routine clinical practice? Implications for enhancing clinical utility in DSM 5.. Psychiat Res.

[11] Bardone-Cone AM, Harney MB, Maldonado CR, Lawson MA, Robinson DP (2010). Defining recovery from an eating disorder: Conceptualization, validation, and examination of psychosocial functioning and psychiatric comorbidity.. Behav Res Ther.

[12] Burgess PW, Shallice T (1997). The Hayling and Brixton Tests.

[13] Nelson HE, Wilson JW (1992).

[14] Wechsler D (1981). Wechsler Adult Intelligence Scale-Revised.

[15] Crawford JR, Deary IJ, Starr JM, Whalley LJ (2001). The NART as an index of prior intellectual functioning: A retrospective validity study covering a 66 year interval.. Psych Med.

[16] Lopez C, Stahl D, Tchanturia K (2010). Estimated IQ in anorexia: A systematic review of the Literature.. Ann Gen Psychia.

[17] Cohen J (1988). Statistical power analysis for the behavioral sciences (2nd ed.).

[18] Holliday J, Tchanturia K, Landau S, Collier D, Treasure J (2005). Is impaired set-shifting an endophenotype of anorexia nervosa?. Am J Psychiatr.

[19] Tenconi E, Santonastaso P, Degortes D, Bosello R, Titton F (2010). Set-Shifting abilities, central coherence, and handedness in anorexia nervosa patients, their unaffected siblings and healthy controls: exploring putative endophenotypes.. World J Bio Psychia.

[20] McAnarney E, Zarcone J, Singh P, Michels J, Welsh S (2011). Restrictive Anorexia Nervosa and Set-Shifting in Adolescents: A Biobehavioral Interface.. J Adolescent Health.

[21] Schmidt U, Treasure J (2006). Anorexia nervosa: Valued and visible. A cognitive-interpersonal maintenance model and its implications for research and practice.. Brit J Clin Psych.

[22] Genders R, Tchanturia K (2010). Cognitive Remediation Therapy (CRT) for Anorexia in Group Format: A Pilot study.. Weight Eat Disord.

